# Correlation of hypothetical virulence traits of two *Streptococcus uberis* strains with the clinical manifestation of bovine mastitis

**DOI:** 10.1186/s13567-015-0268-y

**Published:** 2015-10-23

**Authors:** Riccardo Tassi, Tom N. McNeilly, Anja Sipka, Ruth N. Zadoks

**Affiliations:** Moredun Research Institute, Pentlands Science Park, Bush Loan, Penicuik, EH26 0PZ UK; Department of Population Medicine and Diagnostic Sciences, College of Veterinary Medicine, Cornell University, Ithaca, NY 14853 USA; Institute of Biodiversity, Animal Health and Comparative Medicine, College of Medical, Veterinary and Life Sciences, University of Glasgow, Glasgow, G61 1QH UK

## Abstract

*Streptococcus uberis* is a common cause of clinical and subclinical mastitis in dairy cattle. Several virulence mechanisms have been proposed to contribute to the species’ ability to cause disease. Here, virulence characteristics were compared between *S. uberis* strains FSL Z1-048, which consistently caused clinical mastitis in a challenge model, and FSL Z1-124, which consistently failed to cause disease in the same model, to ascertain whether in vitro virulence characteristics were related to clinical outcome. Macrophages derived from bovine blood monocytes failed to kill FSL Z1-048 whilst reducing survival of FSL Z1-124 by 42.5%. Conversely, blood derived polymorphonuclear cells caused more reduction (67.1 vs. 44.2%, respectively) in the survival of FSL Z1-048 than in survival of FSL Z1-124. After 3 h of coincubation with bovine mammary epithelial cell line BME-UV1, 1000-fold higher adherence was observed for FSL Z1-048 compared to FSL Z1-124, despite presence of a frame shift mutation in the *sua* gene of FSL Z1-048 that resulted in predicted truncation of the *S. uberis* Adhesion Molecule (SUAM) protein. In contrast, FSL Z1-124 showed higher ability than FSL Z1-048 to invade BME-UV1 cells. Finally, observed biofilm formation by FSL Z1-124 was significantly greater than for FSL Z1-048. In summary, for several hypothetical virulence characteristics, virulence phenotype in vitro did not match disease phenotype in vivo. Evasion of macrophage killing and adhesion to mammary epithelial cells were the only in vitro traits associated with virulence in vivo, making them attractive targets for further research into pathogenesis and control of *S. uberis* mastitis.

## Introduction

*Streptococcus uberis* is one of the most common causes of clinical and subclinical mastitis in dairy cattle [1, 2]. Infections may be transient or chronic, associated with low or high somatic cell count (SCC), and limited to an individual animal or part of an outbreak affecting multiple animals [3, 4]. In addition, *S. uberis* may be found in bovine faeces and in the farm environment [5, 6]. Based on whole genome analysis of the type strain O140J, *S. uberis* has been described as an opportunistic pathogen that utilises nutritional flexibility to adapt to a range of ecological niches, including the mammary gland [7]. Others, however, have proposed numerous virulence traits that may be associated with the ability of *S. uberis* to cause mastitis. Examples include production of antiphagocytic factors that affect interaction with macrophages and neutrophils [1, 8], the ability to adhere to and invade into bovine mammary epithelial cells, potentially mediated by the *S. uberis* adhesion molecule SUAM [9, 10], and the ability to form biofilm [11, 12].

Macrophages represent a major component of the cell population in the healthy lactating mammary gland and the first line of defense against pathogens [13–16]. Their general function is to phagocytise and kill pathogens. After intramammary challenge with *S. uberis* strain O140J, microscopic analysis demonstrated presence of the bacteria within the cytoplasm of mammary macrophages. This suggests that macrophages are capable of phagocytising *S. uberis* and may play a role in its clearance from the mammary gland, although actual killing of *S. uberis* O140J was not observed during in vitro experiments [17, 18]. Following the recognition of pathogens via pattern recognition receptors, mammary gland macrophages are involved in the initiation of the immune response. They produce proinflammatory cytokines such as TNF-α and interleukin (IL)-1β and chemokines such as IL-8, which contributes to migration of polymorphonuclear cells (PMN) from the blood stream to the mammary gland [14, 19, 20]. The role of PMN is to ingest and kill the bacteria at the site of the infection. PMN are crucial in response to intramammary infection (IMI) caused by major mastitis pathogens such as *Escherichia coli* and *Staphylococcus aureus* [20–22]. For *S. uberis*, the ability of PMN to kill different strains has been linked to strain-specific virulence. Hill [23] selected two strains with extreme differences in susceptibility to killing by PMNs and showed that the strain that was most resistant to killing, *S. uberis* O140J, had a higher ability to cause clinical mastitis in lactating cows than *S. uberis* EF20, which was least resistant to killing [23]. Separate challenge studies conducted with strain O140J [24] or strain UT888 [25] showed that massive influx of PMN into the mammary gland failed to control *S. uberis* infection. In contrast, a challenge study conducted with *S. uberis* U103 [26] showed that influx of PMN reduced the number of bacteria after an initial peak. Taken together, the observations are consistent with the idea that some strains are more resistant to PMN killing than others, and that this may contribute to differences in clinical manifestation of infections.

In addition to interaction with leucocytes, interaction with host epithelial cells plays a role in the pathogenesis of infection. Adhesion to and internalization into epithelial cells are considered a crucial stage for the infection process in bacterial diseases because they allow the pathogen to target the appropriate tissue [27]. In the context of the mastitis, adherence to mammary gland epithelia would allow the bacteria to colonize the lactating mammary gland despite the flow of milk, which results in excretion of non-adherent bacteria [1]. Invasion of epithelial cells would prevent phagocytes from eliminating the bacteria and may be associated with spread of the infection to deeper tissues and development of persistent infections [2, 28]. Adhesion and invasion into MAC-T cells is in part mediated by the protein *S. uberis* adhesion molecule (SUAM) and antibodies against SUAM reduce the ability of *S. uberis* strains to adhere to and invade MAC-T cells in vitro [10, 29]. SUAM is highly conserved in *S. uberis* strains [30, 31], but their ability to invade mammary epithelial cells is variable [9, 28]. After invasion, bacteria survive and possibly replicate in the epithelial cells up to 120 h after challenge, with different strains doing this at different rates [28]. For *E. coli*, differences in invasion and intracellular survival have been linked to the difference between transient and persistent IMIs [32].

Like invasion of mammary epithelial cells, the role of biofilm formation in *S. uberis* mastitis has only been investigated in vitro. Biofilm is a sessile form of bacterial growth. Bacteria growing as biofilm are described as “a structured community formed by bacteria themselves enclosed in a self-produced polymeric matrix and attached to an inert or living surface” [33]. In the mammary gland, this would allow the bacteria to resist host defences as well as the effect of antimicrobial treatment [34]. Differences in the ability to form biofilm have been found between different strains of *S. aureus*, with strains isolated from the mammary gland more capable of forming biofilm in vitro than strains isolated from extra-mammary sources such as teat skin and milking unit liners [35]. This suggests a possible role for biofilm formation in bacterial colonisation of the mammary gland. Ability to grow as biofilm has also been shown for *S. uberis*, with variation between strains but without a clear correlation between biofilm formation and the ability to cause clinical mastitis [11, 12].

We previously conducted an intramammary challenge study to investigate the host response to two strains of *S. uberis*, resulting in consistent responses across cows and clear differences in virulence between strains, with one strain resulting in clinical mastitis in all cases and the other strain inducing no clinical disease [36]. The ability of the two strains to grow in milk of the challenged animals did not explain the observed difference in virulence, because the non-virulent strain grew faster in milk than the virulent strain [36]. In the current study, we try to explain the difference in virulence that was observed in vivo through further investigation of several putative virulence mechanisms in vitro, including ability to escape killing activity of host phagocytes, adhesion to and invasion of mammary epithelial cells, biofilm formation and presence and composition of the *sua* gene.

## Materials and methods

### Bacteria

Two strains of *S. uberis* were selected to represent different clinical and epidemiological phenotypes as well as distinct genotypes. Strain FSL Z1-048 was originally obtained from a cow with chronic subclinical mastitis in mid-lactation as part of a contagious *S. uberis* mastitis outbreak. Strain FSL Z1-124, isolated around the same time from the same herd, was obtained from a heifer with transient clinical mastitis at calving and was not part of a mastitis outbreak [4]. Based on multilocus sequence typing, which is a standardized method for molecular typing of bacteria [37], the isolates belong to sequence type (ST) 385 and ST383, respectively. ST385 is part of clonal complex 143, which has been linked to subclinical mastitis, whereas ST383 differs from all known sequence types by at least three alleles and does not form part of a clonal complex [3, 37]. In addition, the isolates are genetically distinct by presence or absence of a large number of open reading frames [38]. When used in challenge experiments, FSL Z1-048 consistently induced clinical mastitis in challenged quarters whereas FLS Z1-124 consistently failed to cause clinical mastitis or even IMI [36].

### Monocyte derived macrophage killing assay

The ability of bovine monocyte derived macrophages to kill *S. uberis* FSL Z1-048 and FSL Z1-124 was tested. Cells were obtained from 3 non-lactating Holstein heifers of 9–12 months of age. The experiment was conducted at the Moredun Research Institute (Penicuick, UK) with approval of the Institute’s Experiments and Ethical Review Committee under home office licence 60/4380 in accordance with the Animals (Scientific Procedures) Act 1986.

Approximately 100 mL of blood were collected from the jugular vein of an individual animal and mixed immediately with an equal volume of Alsever’s solution as anticoagulant (d-glucose 113.76 mM, sodium chloride 71.87 mM, sodium citrate dihydrate 27.20 mM, citric acid 2.86 mM in water). Peripheral blood mononuclear cells (PBMC) were isolated by layering the mixture of blood and anticoagulant onto Ficoll-Paque PLUS (GE healthcare, Amersham, UK) at a ratio of 2:1 and the PBMC layer was separated by centrifuging at 900 ×* g* for 30 min at 4 °C. The PBMC layer was pipetted off and transferred to a new falcon tube and washed three times in complete medium (RPMI-1640 supplemented with 10% vol/vol heat inactivated FCS, 200 U/mL penicillin, 200 U/mL streptomycin, 1% vol/vol glutamine; Sigma-Aldrich, Dorset, UK). Cells were finally resuspended in up to 2 mL buffer, then labelled with mouse anti-human CD14 microbeads (Miltenyi Biotec, Bisley, UK) and CD14^+^ cells isolated by positive selection on an LS magnetic column (Miltenyi Biotec) following manufacturer’s instructions. Viable cells were counted by trypan blue dye exclusion and seeded in complete medium at 1 × 10^6^ cells/well in flat-bottom 96-well polystyrene tissue culture plates (Corning, Tewksbury, MA, USA). Cells were cultured for 6 days at 37 °C, 5% CO_2_. Medium was changed every second day. 24 h before the assay, complete medium was replaced with complete medium without antimicrobials. In preliminary experiments, purity of macrophage culture after the magnetic bead separation was assessed by flow cytometry. Briefly, 10 μL of anti-CD14 antibody (clone VPM65, mouse IgG1 isotype, Moredun Research Institute [39]) were added to 1 mL of cell suspension in complete medium containing 1 × 10^6^ cells and incubated for 30 min at 4 °C. Cells were washed three times by centrifugation at 1500 × *g* for 5 min at 4 °C. 100 μL of goat anti-mouse IgG Alexa 488 conjugated secondary antibody diluted 1:2000 were added and incubated for 30 min at 4 °C. Cells were acquired with a 2-laser Cyan flow cytometer (Beckman Coulter, High Wycombe, UK). Data were collected for a minimum of 10000 cells for each sample, and cells were gated based on forward and side scatter to exclude cellular debris from the analysis. Data were analysed using FlowJo software (FlowJo, Ashland, OR, USA). The percentage of CD14 positive cells was calculated. Cytocentrifuge preparations of selected cell samples after 7 days of culture were prepared using a Shandon Cytospin 4 cytocentrifuge (Thermo Electron Corporation, Milford, MA, USA) and stained using a REASTAIN Quick-Diff Kit (Reagena, Toivala, Finland) for microscopic examination.

To conduct the macrophage killing assays, 100 μL of supernatant were removed and replaced with 100 μL of medium containing *S. uberis* FSL Z1-048 or FSL Z1-124, which had been pre-incubated for 20 min at 37 °C with heat inactivated adult bovine serum (Life Technologies; Paisley, UK) as source of opsonins. Based on preliminary experiments, bacteria were diluted to have a multiplicity of infection (MOI) of 5 (i.e. 5 bacteria per cell). Bacteria and cells were co-incubated for 2 h at 37 °C in 5% CO_2_ before 50 μL of 0.1% vol/vol triton X-100 (Sigma-Aldrich) diluted in PBS were added to lyse the cells. Cell lysates were tenfold serially diluted in cold PBS and 20 μL spots were plated in triplicate on blood agar plates (E&O Laboratories, Bonnybridge, UK). Colony forming units (cfu) were counted, where possible for the dilution presenting 5–50 colonies per spot, and concentration was calculated. Each result was based on the average of 3 wells and per strain, 3 biological replicates (i.e. blood from 3 individual animals) and 2 technical replicates (iterations of the assay) were used.

### PMN killing assay

To test the ability of bovine PMN to kill *S. uberis*, PMN were isolated from blood of 4 Holstein cows in mid-lactation (parity 2–5). The experiment was conducted at the Department of Population Medicine and Diagnostic Sciences, College of Veterinary Medicine, Cornell University (Ithaca, NY, USA) using animals held by the Cornell Teaching and Research Facility (Ithaca, NY, USA). All procedures were approved by the Cornell Institutional Animal Care and Use Committee.

Approximately 20 mL of blood were collected from the jugular vein of individual animals in vacutainer vials containing heparin as anti-coagulant. Blood was processed within 1 h of collection. It was diluted with an equal volume of PBS and the mixture was layered on Ficoll-Paque plus (GE Healthcare) and centrifuged for 30 min at 900 × *g* at 4 °C. The granulocyte layer was transferred to a fresh 50 mL falcon tube. Residual erythrocytes were lysed by adding 5 mL of pre-warmed lysis solution (0.8% wt/vol NH_4_Cl, 0.1 mM EDTA in water, 37 °C). Cell suspensions were gently mixed until the erythrocytes were completely lysed. 20 mL of PBS were added, then cells were washed twice by spinning at 300 ×* g* for 10 min at 4 °C and finally resuspended in RPMI-1640 (Life Technologies) supplemented with 10% heat inactivated fetal calf serum (FCS) and 4 mM glutamine. Viable cells were microscopically counted based on trypan exclusion staining. Cells were seeded in 96-well polystyrene cell culture plates (Corning) at 5 × 10^5^ cells per well in a total volume of 200 μL. After pre-incubation for 20 min at 37 °C with heat inactivated adult bovine serum (Life Technologies) as source of opsonins, *S. uberis* FSL Z1-048 or FSL Z1-124 was added to wells at MOI of 1 with PMN. Bacteria and PMN were co-incubated for 90 min at 37 °C, 5% CO_2_. To lyse the cells, 50 μL of 0.1% vol/vol triton X-100 (Sigma-Aldrich) diluted in PBS were added. Cell lysates were tenfold serially diluted in cold PBS and 20 μL spots were plated in triplicate on blood agar plates (BioMerieux Inc. Durham, NC, USA). Cfu were counted, where possible for the dilution presenting 5–50 colonies per spot, and concentration was calculated. In preliminary experiments, purity of the cell population after isolation of PMN was tested with flow cytometry analysis. Cells were stained with antibodies against CD11b (Monoclonal FITC conjugated, Mouse IgG2b, VMRD Inc., Pullman, WA, USA) and anti CD14 (Monoclonal APC conjugated Mouse IgG1, VMRD Inc.) antigens using a protocol described by Schwarz et al. [16]. Each result was based on the average of 3 wells and per strain, 4 biological replicates (i.e. blood from 4 individual animals) were used.

### Mammary epithelial cell adhesion and invasion assay

In mammary epithelial cell invasion assays, *Salmonella enterica* serovar Typhimurium strain 12023 was included as positive control [40] (kindly provided by Professor David GE Smith, Moredun Research Institute), whilst *E. coli* laboratory strain TOP10 (Invitrogen, Paisley, UK) was included as negative control for the invasion assay. *Streptococcus uberis* strains were grown in brain heart infusion broth (BHI; Oxoid, Basingstoke, UK) for 18 h at 37 °C under shaking (200 rpm) whereas the positive and negative control strains were grown in Lysogeny broth (BD, Oxford, UK) for 18 h at 37 °C under shaking (200 rpm). Bacterial suspensions were centrifuged at 4000 ×* g* for 20 min at 4 °C, washed three times in cold PBS, and finally resuspended in PBS. Concentrations of live bacteria were determined by viable count method [36]. Suspensions were diluted to the target concentrations and stored at 4 °C prior to use.

Immortalized bovine mammary epithelial cell line BME-UV1 cells [41] were cultured at 37 °C under 5% CO_2_ in 75 cm^2^ tissue culture flasks (Corning). BME-UV1 complete medium consisted of a mix containing 40% Ham’s F-12 nutrient mixture, 30% RPMI-1640 medium and 20% NCTC 135 medium. All media were obtained from Life Technologies. The medium was supplemented with 10% (wt/vol) heat inactivated FCS, 0.1% (wt/vol) lactose, 0.1% (wt/vol) lactalbumin hydrolysate, 1.2 mM glutathione, 10 μg/mL l-ascorbic acid, 1 μg/mL hydrocortisone, 1 μg/mL insulin, 200 U/mL penicillin and 200 U/mL streptomycin. All reagents were obtained from Sigma-Aldrich. Cells were cultured until 70–80% confluency, as judged visually by light microscopy, and then washed with warm PBS and treated with 1 mL of TrypLE Express dissociation media (Life Technologies) supplemented with 4 U/mL of porcine elastase (Sigma-Aldrich) and incubated at 37 °C until 70–80% of the cells were showing a shrunken morphology. Medium was then discarded and 1 mL of TrypLE Express dissociation medium per flask was added. Flasks were incubated until the cells were completely detached from the plastic surface. Complete medium was added and cells transferred to a new flask. Cells from passage 5–10 were transferred to 24-well tissue culture plates (Corning) at 1 × 10^5^ cells/well and cultured for 1 day in complete medium. Medium was discarded and cells were rinsed three times with warm PBS. The cells were then cultured in antibiotic free medium for 18 h until the assay was performed.

Bacterial challenge preparations were added to the supernatant of the completely confluent monolayer of BME-UV1 cells (approximately 1 × 10^6^ cells/well). *Streptococcus uberis* FSL Z1-048 or FSL Z1-124 was added to obtain a final concentration of 1 × 10^7^ and 1 × 10^8^ cfu/mL in the culture media, representing multiplicity of infection (MOI) of 10 and 100 (bacteria:epithelial cell ratio) respectively. Positive and negative controls were added at approximately MOI of 1. Cells and bacteria were co-incubated for 3 h at 37 °C under 5% CO_2_. Cell culture supernatants were discarded and wells were vigorously washed three times with warm PBS to eliminate non-adherent bacteria. Cells were lysed by adding 500 μL per well of 0.1% vol/vol Triton X-100 (Sigma-Aldrich) diluted in PBS. Lysates were serially diluted in cold PBS and 20 μL spots of the serial dilutions were plated in triplicate on blood agar plates (E&O Laboratories). Plates were incubated overnight at 37 °C, colonies were counted and the concentration of bacteria (cfu/mL) associated with the cells (adhered to the surface and internalized) was calculated. In parallel assays, performed on separate plates, washed cells were incubated for 1 h at 37 °C, 5% CO_2_ with 1 mL per well of growth medium supplemented with 150 μg/mL gentamicin to kill the extracellular bacteria so that internalization but not adherence was measured. Cells were washed 3 times with warm PBS and lysed by adding 500 μL per well of 0.1% vol/vol Triton X-100 (Sigma-Aldrich) diluted in PBS. Lysates were serially diluted in cold PBS and 20 μL spots of the serial dilutions were plated in triplicate on blood agar plates (E&O Laboratories). Plates were incubated overnight at 37 °C, colonies were counted and the concentration of bacteria (cfu/mL) internalized in the cells was calculated. The number of bacteria that adhered on the surface was obtained by subtracting the concentration of internalized bacteria from the total concentration of bacteria associated with the cells [28]. The experiment was conducted three times, with the results for each strain based on the average of three wells in each replicate.

### Biofilm assay

The ability to form biofilm was assessed using FSL Z1-048, FSL Z1-124 and a positive control (*S. uberis* strain 20539) in a protocol described by Gilchrist [42] with some modifications. *Streptococcus uberis* strains were cultured in BHI for 16 h at 37 °C under shaking (150 rpm). BHI culture was diluted 100-fold in one of three different media: (1) BME-UV1 complete medium, (2) BME-UV1 complete medium supplemented with 0.5% wt/vol casein, and (3) RPMI-1640 chemically defined medium (CDM) supplemented with 0.5% casein. RPMI-1640 CDM was composed of RPMI-1640 (Sigma-Aldrich) supplemented with 0.8% wt/vol lactose, 260 mg/L l-glutamic acid, 150 mg/L magnesium sulphate heptahydrate, 10 mg/L ferrous sulphate heptahydrate, 10 mg/L manganese sulphate tetrahydrate. All reagents were obtained from Sigma-Aldrich. Two hundred μL per well of inoculated media were aliquoted into 96-well flat bottom polystyrene tissue culture plates (Corning). Plates were incubated statically at 37 °C in 5% CO_2_ for 24 h. Medium was discarded and wells were washed three times with distilled water to remove cells not adhered to the plate. Plates were air dried for 45 min and biofilms were stained by adding 100 μL/well of 1% crystal violet solution (Fisher Scientific, Loughborough, UK). Stain was discarded and wells washed with distilled water until excess stain was completely removed. Plates were air dried for 45 min, followed by addition of 200 μL/well of ethanol and incubation for 15 min. Optical density (OD) at 562 nm was read with an ELx808 absorbance microplate reader (Biotek instruments, Potton, UK) to quantify biofilm formation. Sample to positive (S/P) ratio for each strain was calculated by dividing the difference between average OD for that strain and average negative control OD by the difference between the average positive control OD and average negative control OD. For RPMI-1640 CDM supplemented with 0.5% casein, 3 biological replicates were performed whereas the biofilm formation in BME-UV1 complete medium and BME-UV1 complete medium with 0.5% casein was tested once based on results from the first round of assays (see Results). The experiment was conducted three times, with results based on the average of eight wells per strain in each replicate.

### *Sua* sequencing

The 2970 bp nucleotide sequence of the *sua* gene was amplified using the forward primer LfbpDL5 5′-GTCATTTGGTAGGAGTGGCTG-3′ and the reverse primer LfbpDL6 5′-TGGTTGATATAGCACTTGGTGAC-3 [30] which provide full length amplification of the gene. PCR was conducted in a final volume of 100 μL containing 50 μL of GoTaq Green master mix (Promega, Madison, WI, USA), 50 μL of water, 300 nM of primers LfbpDL5 and LfbpDL6 and 100 ng of genomic DNA as template. The cycling profile consisted of 94 °C for 2 min followed by 30 cycles of 30 s at 94 °C, 30 s at 54 °C, and 2 min at 68 °C followed by 7 min at 68 °C. PCR product (15 μL) was visualized on a 1% agarose gel. The PCR products were purified using wizard SV gel and PCR clean-up system (Promega, Southampton, UK). Two 15 μL aliquots of the purified PCR products were sent to Eurofins Genetic Services (London, UK) for bidirectional sequencing using the following forward primers: LfbpDL5, 5′-TCAAATGCCTC-3′, 5′-CCGTATCGTTAACCCAGA-3′ and 5′-CGCTTGTTCACCTAA ATCAG-3′. The following primers were used in the reverse direction: LfbpDL6, 5′-CGCTTCTTGAGCAGTATCTACTG-3′, 5′-GACTGATTTAGGTGAACAAGCG-3′ and 5′-CTGGGTTAACGATACGG-3′ [30]. Sequence reads were aligned, checked for quality of the electopherogram and analysed using the SeqMan program within the Lasergene 11 package (DNASTAR, WI, USA). *Sua* nucleotide sequences were aligned with the reference sequence of strain UT888 (GenBank: DQ232760.1). The nucleotide sequence of *sua* was further analysed using sequence manipulation suite ORF finder [43] to assess the presence of stop codons and determine the translated amino acid sequences, and submitted to the European Nucleotide Archive.

### Statistical analysis

Statistical analyses were conducted in Genstat (VSN International, Hemel Hempstead, UK). For all statistical analyses, significance was declared when *p* < 0.05. For macrophage and PMN killing assays, percentage bacterial survival was calculated as the proportion of viable bacteria in wells with phagocytes compared to control wells containing bacteria and growth medium only. Percentage survival was compared between strains using a t test. Adhesion and invasion data, expressed as concentration of bacteria that invaded cells and concentration of bacteria that adhered to the cells, respectively, were base-10 logarithmically transformed to ensure the data from each treatment group had an approximately normal distribution. The log-transformed concentrations were used as outcome variable in a two-way ANOVA with strain and MOI as treatment factor. Replicate number was used as blocking factor. The interaction between strain and MOI was also evaluated. Normality of the distribution of the residuals was assessed using Q–Q probability charts with 95% confidence limits and by the Anderson–Darling marginal test. Homogeneity of variance of the residuals was assessed by Bartlett’s test. Post-hoc comparisons with Tukey HSD test were performed where appropriate. Results from the biofilm experiments were not normally distributed and results were analysed by non-parametric Mann–Whitney test, using S/P as input to correct for potential plate-to-plate variability.

## Results

### Macrophage killing assay

**Figure 1:**
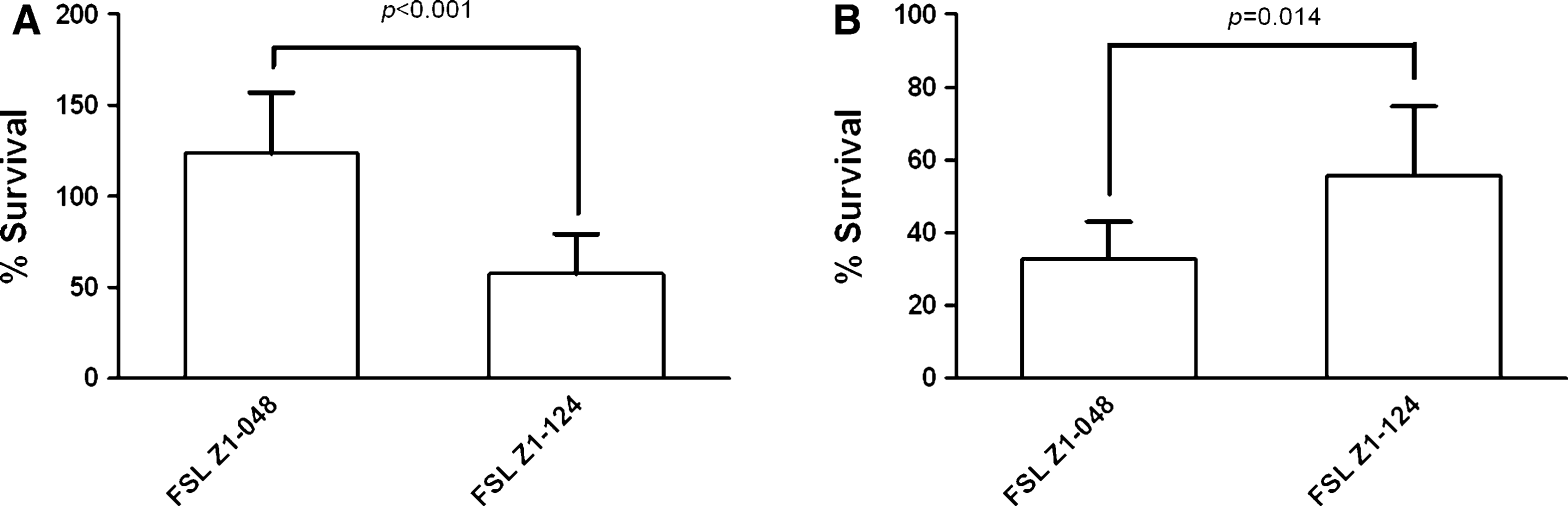
**Uptake and killing of** ***Streptococcus uberis*** **by host phagocytes.** **A** Killing of *S. uberis* strains FSL Z1-048 and FSL Z1-124 by bovine monocyte derived macrophages. Data represent the percentage (±standard deviation) of bacteria that survived after 2 h of co-incubation with macrophages. Data were obtained using cells isolated from blood of 3 animals, tested individually, and the experiment was conducted twice. The *p* value for the t test is shown, calculated using 6 observations per strain with each observation based on the average of 3 wells. **B** Killing of *S. uberis* strains FSL Z1-048 and FSL Z1-124 by bovine polymorphonuclear cells (PMN). Data represent the percentage (±standard deviation) of bacteria that survived after 1.5 h of co-incubation with PMN. Data were obtained in one experiment using PMN from 4 animals, tested individually. The *p* value for the t test is shown, calculated using 4 observations per strain, with each observation based on the average of 3 wells.

The ability of macrophages derived from blood monocytes to kill *S. uberis* FSL Z1-048 and FSL Z1-124 was tested. Flow cytometry results from preliminary experiments showed that, after purification from blood, approximately 90% of cells were CD14^+^ (results not shown). After 7 days of culture, approximately 95% of cells showed the morphology of differentiated macrophages at microscopic examination. Bactericidal ability of monocyte-derived macrophages was significantly different between the two strains (*p* < 0.001). Monocyte-derived macrophages were able to kill strain FSL Z1-124. The proportion of viable bacteria present after 2 h of co-incubation with host cells was 58 ± 22% of the bacteria incubated in the medium only (Figure 1A). In contrast, macrophages failed to kill strain FSL Z1-048. Bacteria recovered after 2 h were 124 ± 33% of the bacteria incubated in the medium only, suggesting a replication in presence of macrophages (Figure 1A).

### PMN killing assay

Flow cytometry analysis showed that, after the PMN isolation process, approximately 75% of cells were CD11b^+^ CD14^−^ and thus likely to be PMN. This proportion was confirmed by microscopic observation of morphology of cells present in the cell preparations. PMN from bovine blood were able to kill both *S. uberis* strains (Figure 1B). Bactericidal ability of PMN was significantly different between the two strains (*p* = 0.014), with higher bactericidal activity observed against strain FSL Z1-048. Percentage survival for strain FSL Z1-048 was 33 ± 10% whereas for strain FSL Z1-124 it was 56 ± 19%.

### Adhesion and invasion assay

**Figure 2:**
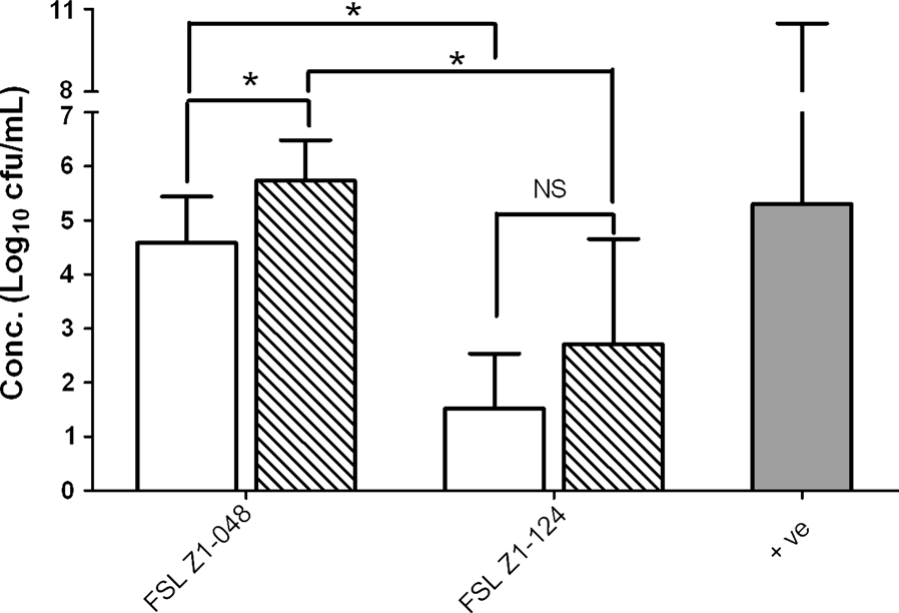
**Adhesion of** ***Streptococcus uberis*** **to bovine mammary epithelial cells BME-UV1 in vitro.** Cells were incubated with strain FSL Z1-048 or strain FSL Z1-124 at 1 × 10^7^ cfu/mL and 1 × 10^8^, to obtain a multiplicity of infection (ratio bacteria:cells) of 10 (white columns) and 100 (striped columns), respectively. Results are expressed as concentration of bacteria recovered after 3 h of co-incubation. The values represent the mean and the standard deviation of 3 replicates, with each replicate based on the average of 3 wells. The solid grey bar represents the concentration of *Salmonella enterica* serovar Typhimurium strain 12023 used as positive control. Replicate number was used as blocking factor. Statistically significant and non-significant differences between groups are marked with asterisks and NS respectively (Tukey HSD test after ANOVA, *p < 0.05).

Both *S. uberis* strains were able to adhere to the bovine mammary epithelial cell line BME-UV1 after 3 h of co-incubation. FSL Z1-048 showed 1000-fold higher levels of adherence than strain FSL Z1-124 at MOI 10 and 100 (*p* < 0.05) (Figure 2). For both strains, adherence increased approximately 15-fold at MOI 100 compared to MOI 10, but the difference in adherence between MOIs was significant only for strain FSL Z1-048 (*p* < 0.05) (Figure 2).

**Figure 3:**
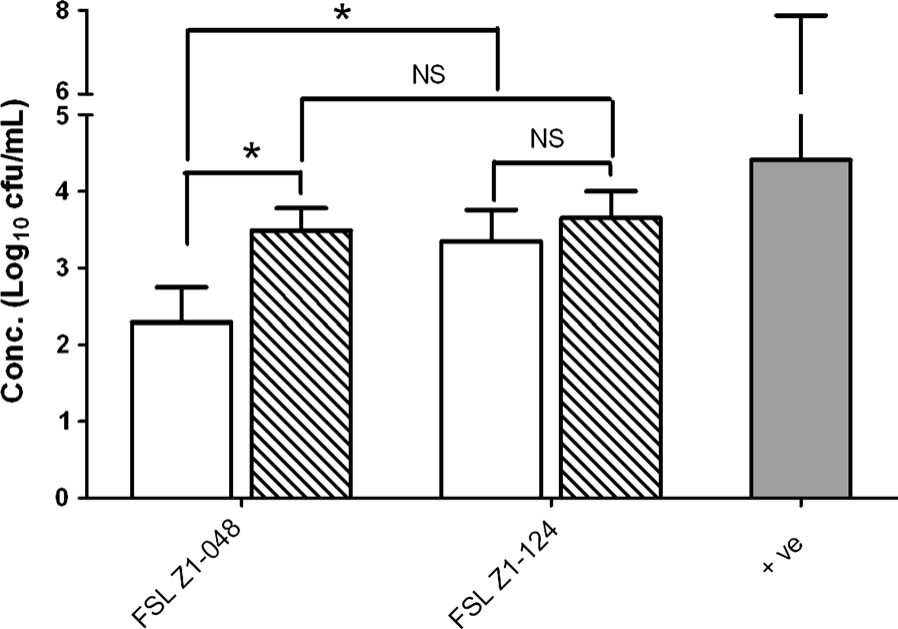
**Invasion of** ***Streptococcus uberis*** **into bovine mammary epithelial cells BME-UV1 in vitro.** Cells were incubated with strain FSL Z1-048 or strain FSL Z1-124 at 1 × 10^7^ cfu/mL and 1 × 10^8^, to obtain a multiplicity of infection (ratio bacteria:cells) of 10 (white columns) and 100 (striped columns) respectively. Results are expressed as concentration of bacteria internalized after 3 h of co-incubation. The values represent the mean and the standard deviation of 3 replicates, with each replicate based on the average of 3 wells. The solid grey bar represents the concentration of *Salmonella enterica* serovar Thypimurium strain 12023 used as positive control. Statistically significant and non-significant differences between groups are marked with asterisks and NS respectively, with replicate used as blocking factor (Tukey HSD test after ANOVA, *p < 0.05).

Both strains were able to invade epithelial cells after 3 h of co-incubation (Figure 3). FSL Z1-124 was 37-fold as invasive as strain FSL Z1-048 at MOI 10 (*p* < 0.05) whereas at MOI 100, invasion was not significantly different between strains. Bacterial concentration had an effect on internalization of strain FSL Z1-048, which was 63-fold as invasive at MOI 100 as at MOI 10 (*p* < 0.05), whereas for strain FSL Z1-124, internalization at MOI 100 was not significantly different to that observed at MOI 10 (Figure 3). The positive control included in the invasion assay, *S. enterica* serovar Typhimurium, was able to adhere to (Figure 2) and to invade (Figure 3) the mammary epithelial cells in all assays. The negative control *E. coli* laboratory strain was not able to invade the cells in any assay (data not shown).

### Biofilm assay

**Figure 4:**
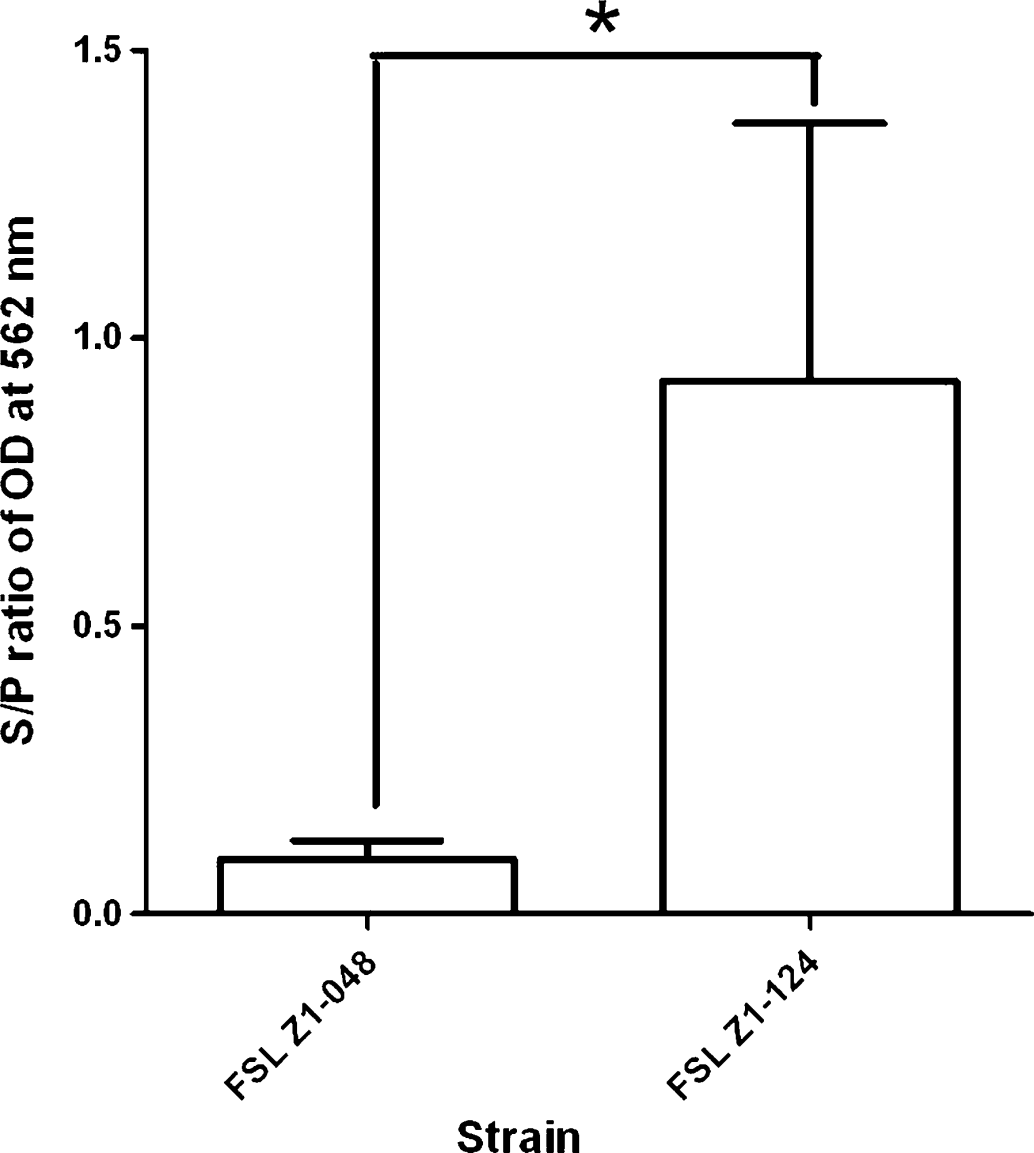
**Biofilm formation of** ***Streptococcus uberis*** **strains FSL Z1-048 and FSL Z1-124.** Biofilm formation was tested in RPMI-1640 CDM medium supplemented with 0.5% wt/vol casein. Strain 20539 was included as positive control (+ve). Bars represent the sample-to-positive (S/P) ratio after 24 h of culture and standard deviations. S/P = (average sample OD − average negative control OD)/(average positive control OD − average negative control OD), where OD = optical density. The assay was conducted on 3 cultures of each strain and for each assay the result was based on the average of 8 wells. Statistically significant difference between the two strains is marked with asterisk (Mann–Whitney test, *p < 0.05).

Ability to form biofilm when grown in RPMI-1640 CDM supplemented with 0.5% casein differed between strains. The ability of FSL Z1-048 to form biofilm was significantly lower (*p* < 0.05; Figure 4) than the ability of strain FSL Z1-124. In preliminary experiments, ability to form biofilm was also tested in BME-UV1 complete medium and BME-UV1 complete medium supplemented with casein but none of the strains were able to form biofilm in these media (results not shown).

### *Sua* sequence

**Figure 5:**
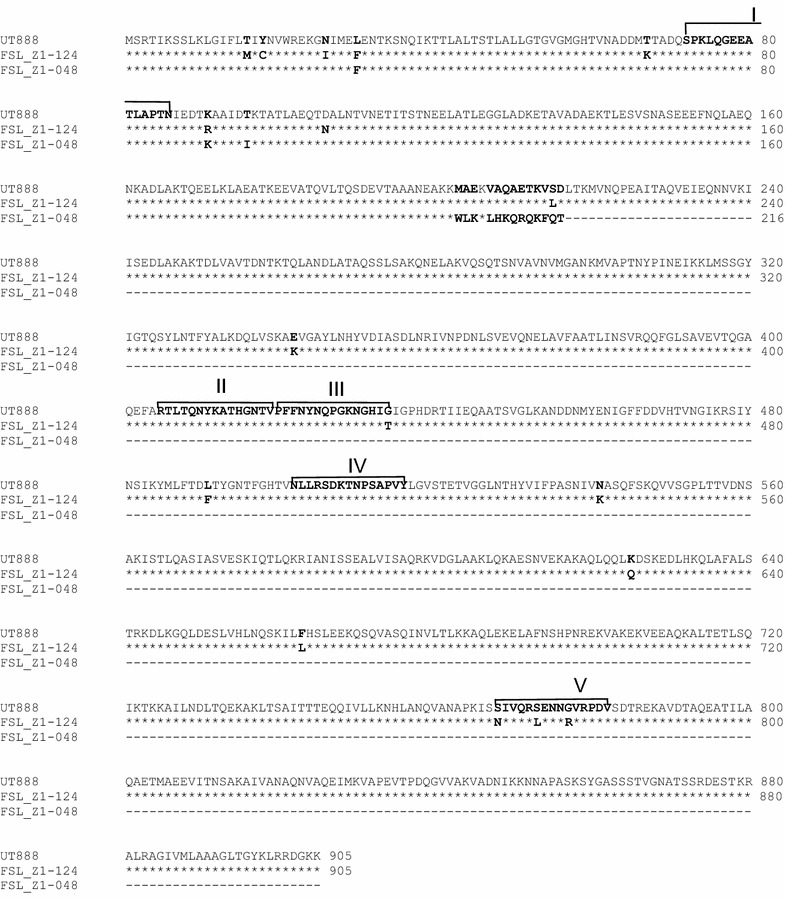
**Alignment of amino-acid sequences of** ***Streptococcus uberis*** **adhesion molecule (SUAM).** Alignment based on translation of *sua* sequences for strains FSL Z1-124, FSL Z1-048, and UT888, showing the five functional domains identified in UT888 (I–V) [28]. Due to a frameshift mutation, the predicted protein of strain FSL Z1-048 is truncated although pep-SUAM is predicted to be intact.

Analysis of open reading frames (ORF) in the full length 2971 bp *sua* gene showed that strain FSL Z1-124 has an ORF extending from base 94 to base 2811 with 99% DNA sequence identity to the ORF from strain UT888 (2939/2971 bp; NCBI Accession number DQ232760.1). The *sua* sequence [European Nucleotide Archive: LN885239] translates into a protein of 905 amino acids in length (Figure 5), which is the full length of the SUAM protein [29]. The protein sequence showed 98% identity with the SUAM protein from strain UT888 (884/905 amino acids). Identity of the *sua* sequence of strain FSL Z1-048 with *sua* of *S. uberis* strain UT888 was 99% (2955/2971), with a one-base pair gap causing a frame-shift mutation in position 700 (5′→3′) of the submitted sequence (European Nucleotide Archive: LN885238). The nucleotide base missing in *sua* of strain FSL Z1-048 resulted in an ORF extending from base 94 to base 744 that would be translated in a truncated SUAM protein of 216 amino acids in length (Figure 5). The sequence encoding pep-SUAM [10] was intact in both strains and the predicted amino acid sequence for FSL Z1-048 was identical to that of UT888, whilst the amino acid sequence for FSL Z1-124 differed by one amino acid.

## Discussion

Different strains of *S. uberis* are associated with different clinical and epidemiological phenotypes [4, 36]. In the present study, we compared a clinically virulent, putatively host-adapted outbreak strain with a non-virulent, opportunistic non-outbreak strain with regards to characteristics that are thought to be associated with virulence, i.e. evasion of host phagocytes, interaction with mammary epithelial cells, biofilm formation and the *sua* gene. Previously, the ability of the two strains to grow in milk had been evaluated [36]. For 5 of 7 hypothetical virulence characteristics, our observations did not match expectations, i.e. the strain that was non-virulent in vivo displayed the more virulent phenotype or genotype in vitro (Table 1). The strain that was virulent in vivo had a more virulent phenotype in vitro only for evasion of macrophage killing and adhesion to mammary epithelial cells. This direct comparison of strains with clearly described clinical and epidemiological phenotypes casts doubt on the biological importance of some supposed virulence traits, including presence of an intact *sua* gene. Although the heterogeneity of the *S. uberis* population necessitates further studies to determine whether these observations extend to other strains, these result indicate which traits may deserve to be prioritised in further research into the pathogenesis and control of *S. uberis* mastitis. It is also possible that different strains use different combinations of virulence traits, and that there is no single mechanism explaining virulence, as recently suggested based on genomic studies [44].

**Table 1:** Overview of potential virulence characteristics of *Streptococcus uberis* and their in vitro manifestation in FSL Z1-048 and FSL Z1-124, which were virulent and non-virulent, respectively, in experimental challenge studies [36]

Virulence trait	FSL Z1-048	FSL Z1-124
Growth in milk^a^	↓^b^	↑
Resistance to macrophage killing	↑	↓
Resistance to PMN killing	↓	↑
Adhesion	↑	↓
Invasion	↓	↑
Biofilm formation	↓	↑
*Sua*	↓	↑

^a^Based on Tassi et al. [36]

^b^↑ Means that the trait as observed in vitro would be expected to increase virulence in vivo compared to the other strain and ↓ means that the trait as observed in vitro would be expected to decrease virulence in vivo.

Macrophages are one of the most common cell populations in the healthy lactating mammary gland and are thought to be the first defence in case of infection [14, 15]. In our study, bovine macrophages were able to kill the non-virulent *S. uberis* strain in vitro whereas they were not able to kill the virulent strain. If in vitro results are representative of macrophage activity in vivo, resident macrophages may be able to clear the non-virulent bacteria before infection becomes established. Conversely, virulent strain FSL Z1-048 would not be cleared by macrophages and thus bacteria would be able to replicate within the mammary gland. Failure to kill *S. uberis* by bovine milk macrophages was similarly observed in a previous study of the clinically virulent strain O140 J [18]. Although different methods were used in the different studies, the results suggest that the ability to resist killing by macrophages may play a role in the pathogenicity of *S. uberis*. Results from our challenge study showed that differences in pathogenesis exist very early in the infection process [36]. Within 18 h after challenge, a pronounced difference in bacterial numbers was seen between the virulent strain and the non-virulent strain, whilst differences were not observed yet in clinical parameters, somatic cell count or cytokine levels at that time. This difference in abundance in the first 18 h after challenge was not explained by the ability of the strains to grow in milk, with the non-virulent strain growing faster in the challenge cows’ milk in vitro than the virulent strain [36]. Thus, the in vitro and in vivo data support the idea that resident macrophages, as the first line of defence in the mammary gland, are at least in part responsible for strain-specific results early after intramammary challenge with *S. uberis*.

In vitro, bovine PMN were able to kill both FSL Z1-048 and FSL Z1-124. Surprisingly, the virulent strain was killed more easily than the non-virulent strain. This result is in contrast to that obtained by Hill, who observed that the ability to resist PMN killing was related with the virulence of the strain when infused into the lactating mammary gland [23]. PMN are present in relatively low numbers in the healthy mammary gland and they are recruited in large numbers from the blood stream following an inflammatory stimulus [19]. This was observed in our challenge study at 30–42 h post-challenge. By that time, bacterial numbers and clinical outcome were already vastly different between strains, so strain-specific virulence during onset of infection was not driven by the interaction with PMNs. PMN may, however, play a role in the resolution of infection as massive influx of PMN coincided with the onset of a 10000-fold reduction in cfu count [36]. At that time, levels of various cytokines were also significantly increased compared to baseline levels, which may enhance the ability of PMN to kill bacteria in vivo.

Adhesion to and invasion of epithelial cells of the mammary gland have been suggested to be a crucial step in development of IMI by *S. uberis* [9, 28]. In vitro, both strains tested in our study were able to adhere to UV-BME cells after 3 h of co-incubation. Adherence was affected by MOI, but at both MOI levels tested, the virulent strain had a higher ability to adhere to mammary epithelial cells than the non-virulent strain. In the challenge study, cows were first milked at 9 h post challenge. Based on growth assays in milk of the challenge cows, bacterial concentrations at 9 h post inoculation were higher for the non-virulent strain than for the virulent strain [36]. If, however, the non-virulent strain did not adhere well to epithelial cells, most of the bacteria may have been removed at milking. Conversely, the virulent bacteria, although potentially lower in number based on growth assays, may have adhered and persisted in the mammary gland during milking. Together with killing of the non-virulent strain by macrophages, the difference in adherence may help to explain why cfu counts were so different between the two strains during experimental challenge even before PMN influx, clinical signs or cytokines were observed. These results also suggest that key early events during colonisation had the largest impact on the subsequent pathogenicity of the two strains.

Both strains were able to invade mammary epithelial cells but the non-virulent strain, FSL Z1-124, showed more invasion at lower MOI. The ability of FSL Z1-124 to invade epithelium did not change with MOI, whereas the invasive ability of FSL Z1-048 was greater at higher MOI. An increase in bacterial concentration, as observed in vivo [36], could increase the ability of this strain to invade the mammary epithelium after the initial stage of the infection. Internalization in mammary epithelial cells would protect the pathogen from phagocytes in the mammary gland. In could also provide protection against antimicrobials used in the therapy of IMI, although some antimicrobials penetrate intracellularly and are effective in the intracellular environment [45]. Invasion of mammary gland cells by *S. uberis* has been observed in vitro but not in challenge studies and its role in vivo remains to be confirmed [17, 26]. In vivo, FSL Z1-124 was rarely and intermittently isolated from challenged quarters, mostly from 12 to 48 h post challenge [36]. In one cow, it was detected in milk at 168 h post challenge, with no positive culture results between 48 and 168 h (unpublished data). Strain identity was confirmed using PFGE [36] to ensure that the positive culture at 168 h was not due to a different strain. The observation of intermittent shedding of FLS Z1-124 could potentially be explained by intracellular survival. Based on our observations, we would like to propose the following hypothetical scenario: after challenge, FSL Z1-124 grows rapidly in milk but it is eliminated by macrophages and, due to its poor adherence to epithelial cells, also by milking. When FSL Z1-124 does adhere, this is followed by rapid invasion into the mammary epithelial cell, where it may survive for several days, as described for other strains in vitro [28], explaining intermittent shedding. FSL Z1-124 was originally isolated from a heifer at calving, demonstrating that it has the ability to cause mastitis, but possibly only during immunosuppression of the host, such as occurs around parturition [46].

In previous studies conducted in vitro, internalization was shown to be partially mediated by *S. uberis* adhesion molecule (SUAM), with deletion of *sua* reducing the ability of *S. uberis* to adhere to and internalize in mammary epithelial cells [47]. The gene encoding SUAM is conserved in strains of *S. uberis* from different geographical locations [30]. Analysis of the full genome sequence of our study isolates showed the presence of *sua* in both strains and suggested the existence of a frame-shift mutation in *sua* of FSL Z1-048 (data not shown). PCR and Sanger sequencing, as reported here, confirmed that the *sua* gene in FSL Z1-124 would code for a protein of 905 amino acids in length as described by Luther et al. [30] whereas *sua* in FSL Z1-048 is predicted to code for a truncated protein of 216 amino acids. Antibodies against pep-SUAM reduce adherence of *S. uberis* to MAC-T cells, demonstrating a role of pep-SUAM in adhesion in vitro [10]. pep-SUAM is predicted to be expressed by FSL Z1-048 and may be functionally active. However, 4 other predicted epitopes thought to be involved in the process of adhesion and invasion mediated by SUAM are located between amino acid residues 342 and 719 [48] and not expressed in FSL Z1-048. Despite this, FSL Z1-048 demonstrated high levels of adherence to UV-BME cells. Recent studies suggest that SUAM is not the only adhesin involved in the adhesion and invasion of mammary epithelial cells as deletion of *sua* reduced but did not eliminate the ability of *S. uberis* to adhere to and invade MAC-T cells in vitro [47]. In addition, assays with random mutants revealed that other adhesins could be involved in the adhesion and invasion process [49]. Therefore, adherence and invasion of strain FSL Z1-048 to the mammary epithelium is likely to be mediated by other, as yet unidentified, SUAM-independent mechanisms. Adherence and invasion were MOI-dependent for FSL Z1-048 but not for FSL Z1-124, suggesting that the underlying mechanism differs between the two strains. For *E. coli¸* as for *S. uberis*, adherence and invasion differ between strains, with some strains showing MOI-dependent invasion into mammary epithelial cells whilst others do not [50, 51].

In the current study, the virulent *S. uberis* strain showed poor biofilm formation whereas the non-virulent strain did form biofilm. The results of the biofilm assay depended strongly on the culture conditions, as exemplified by the lack of biofilm formation in BME-UV1 medium. This implies that observations in the adherence and invasion assays, which were conducted in BME-UV1 medium, will not have been skewed by differences in biofilm formation. Addition of casein, which is thought to be a major inducer of biofilm formation [12], did not affect growth in RPMI-1640 CDM. Differences in biofilm formation under different conditions have previously been described for the reference strain O140J, which showed good biofilm formation in one study and poor biofilm formation in another [11, 12]. It is largely unknown which in vitro conditions would be an appropriate or relevant reflection of in vivo conditions with regards to biofilm formation.

In summary, using two clinically and epidemiologically well-characterised strains of *S. uberis*, we demonstrated strain-dependent differences in the resistance against macrophage killing, which may play an important role in the early pathogenesis of IMI. Additionally or alternatively, adhesion to the mammary epithelial cells may help to determine the outcome of intramammary challenge. Thus, these virulence factors represent attractive targets for further research into pathogenesis and control of *S. uberis* mastitis. An intact *sua* gene does not appear necessary for adherence. Growth in milk, phagocytosis by PMN, ability to invade the mammary epithelial cells and biofilm formation in vitro were not associated with virulence differences in vivo although PMN do appear to make an important contribution to elimination of infection. Given the heterogeneity of the *S. uberis* population, further work is required to determine whether these observations extend to other strains.
